# Do monocular myopia children need to wear glasses? Effects of monocular myopia on visual function and binocular balance

**DOI:** 10.3389/fnins.2023.1135991

**Published:** 2023-03-22

**Authors:** Aiqun Xiang, Kaixuan Du, Qiuman Fu, Yanni Zhang, Liting Zhao, Li Yan, Dan Wen

**Affiliations:** ^1^Eye Center of Xiangya Hospital, Central South University, Changsha, Hunan, China; ^2^Hunan Key Laboratory of Ophthalmology, Changsha, Hunan, China; ^3^National Clinical Research Center for Geriatric Disorders, Xiangya Hospital, Central South University, Changsha, Hunan, China; ^4^National Engineering Research Center for Healthcare Devices, Guangzhou, China

**Keywords:** monocular myopia, visual function, binocular balance, stereopsis, accommodation

## Abstract

**Objective:**

This study aims to compare the binocular visual functions and balance among monocular myopic adolescents and adults and binocular low myopic adolescents and explore whether monocular myopia requires glasses.

**Methods:**

A total of 106 patients participated in this study. All patients were divided into three groups: the monocular myopia children group (Group 1 = 41 patients), the monocular myopia adult group (Group 2 = 26 patients) and the binocular low myopia children group (Group 3 = 39 patients). The refractive parameters, accommodation, stereopsis, and binocular balance were compared.

**Results:**

The binocular refractive difference in Group 1, Group 2, and Group 3 was −1.37 ± 0.93, −1.94 ± 0.91, and −0.32 ± 0.27 D, respectively. Moreover, uncorrected visual acuity (UCVA), spherical equivalent (SE) and monocular accommodative amplitude (AA) between myopic and emmetropic eyes in Group 1 and Group 2 were significantly different (all *P* < 0.05). There was a significant difference in the accommodative facility (AF) between myopic and emmetropic eyes in Group 2 (*t* = 2.131, *P* = 0.043). Furthermore, significant differences were found in monocular AA (*t* = 6.879, *P* < 0.001), binocular AA (*t* = 5.043, *P* < 0.001) and binocular AF (*t* = −3.074, *P* = 0.003) between Group 1 and Group 2. The normal ratio of stereopsis according to the random dots test in Group 1 was higher than in Group 2 (χ2 = 14.596, *P* < 0.001). The normal ratio of dynamic stereopsis in Group 1 was lower than in Group 3 (χ2 = 13.281, *P* < 0.001). The normal signal-to-noise ratio of the binocular balance point in Group 1 was lower than Group 3 (χ2 = 4.755, *P* = 0.029).

**Conclusion:**

First, monocular myopia could lead to accommodative dysfunction and unbalanced input of binocular visual signals, resulting in myopia progression. Second, monocular myopia may also be accompanied by stereopsis dysfunction, and long-term uncorrected monocular myopia may worsen stereopsis acuity in adulthood. In addition, patients with monocular myopia could exhibit stereopsis dysfunction at an early stage. Therefore, children with monocular myopia must wear glasses to restore binocular balance and visual functions, thereby delaying myopia progression.

## 1. Introduction

Myopia is a global public health problem (Li et al., 2016). In recent years, the incidence of myopia has increased, and the age of onset has become younger. Monocular myopia is common in school-aged children. Moreover, an inter-ocular difference of 1.00 D or more in cycloplegic spherical equivalent (SE) was considered anisometropia (Afsari et al., 2013; Hu et al., 2016). Myopia changes the refractive status, structure and function of the eyes (Mitchell and Sengpiel, 2009; Sengpiel, 2011). Due to the difference in refractive power between the two eyes, anisometropia will result in different retinal image sizes in each eye, resulting in dysfunctional monocular and binocular vision (Huang et al., 2011). Monocular myopic patients usually do not wear glasses because of their good monocular vision. Does the uncorrected monocular myopia affect the balance between two eyes? This is one of the issues we explored.

Moreover, the visual function of monocular myopia is frequently disregarded. In clinical settings, stereopsis and accommodative functions are normally used to evaluate binocular visual functions (Wilson, 2017; Niechwiej-Szwedo et al., 2020). However, the random dots stereopsis is used to detect only close-range and static stereopsis, limiting the evaluation and accuracy of stereopsis. Therefore, we applied the virtual reality platform to test the binocular vision functions of patients from different dimensions. We employed methods mentioned earlier to observe the changes in visual functions of children and adults with monocular myopia and guide monocular myopia patients on whether they need glasses.

## 2. Materials and methods

### 2.1. Patients

A total of 106 patients were enrolled in the Laser Center of Ophthalmology, Xiangya Hospital of Central South University, from April 2021 to December 2021. The exclusion criteria were as follows: strabismus, amblyopia, organic and congenital ophthalmopathy, nystagmus, history of ocular trauma and surgery. Patients were divided into three groups, 41 children with monocular myopia (Group 1: 23 males and 18 females), 26 adults with monocular myopia (Group 2: 9 males and 17 females), and 39 children with binocular low myopia (Group 3: 21 males and 18 females). Specific inclusion criteria were as follows: in the monocular myopia group, the spherical correction of one eye was −0.5 to −3.00 D, the cylinder was less than −1.50 D, the best corrected visual acuity was 20/20 or better, and the naked eye visual acuity of the other eye was 20/20 or better, the difference of SE between two eyes was greater than or equal to 1.00 D. In binocular low myopia group, the spherical correction ranged from −0.5 to −3.00 D and the cylinder was less than −1.50 D, the best corrected visual acuity of both eyes was 20/20 or better, the difference of SE between two eyes was less than 1.00 D. Children and adults with monocular myopia do not routinely wear glasses, whereas children with binocular low myopia do. Informed consent was obtained from the patients and the parents or legal guardians of the underaged patients. All study protocols were approved by the Medical Ethics Committee of Xiangya Hospital of Central South University and carried out in adherence to the Declaration of Helsinki regarding ethical principles for research involving human subjects.

### 2.2. Measurement of refractive parameters

Cycloplegic eye drops (atropine twice daily) was given for 1 week for children under 8 years. Compound tropicamide eye drops were administered for patients over 8 years, once every 10 min for four times. Computer optometry, ophthalmoscopy combined with subjective refraction to determine spherical and cylinder.

### 2.3. Measurement of accommodative functions

#### 2.3.1. Accommodative amplitude

The AA was measured using Donders’ push-up method. Patients were instructed to focus on the line second from the bottom on a reduced vision chart at a distance of approximately 40 cm and indicate when the target blurred as the chart moved slowly toward the eye. The distance from the target to the spectacle plane was measured with a millimeter ruler and converted to diopters. During monocular measurements, the untested eye was covered with an occluder.

#### 2.3.2. Accommodative facility

The AF was measured with a ±2.00 D flipper. The participants were instructed to read each of the 20/30 letters in order immediately after recognition. First, through the −2.00 DS lens and then the +2.00 DS lens, the number of flips per minute was recorded and converted to cycles per minute (cpm). During monocular measurement, the untested eye was covered with an occluder.

### 2.4. Random dots stereopsis

According to the patients’ daily refractive correction status, monocular myopia patients were tested without glasses, and low myopia patients were tested with glasses. Subjects were measured at a distance of 40 cm. There are two inspection boards, each with four stereoacuity inspection pictures, decreasing from 800–40 arcsec in order of parallax. The result less than or equal to 60 arcsec is normal.

### 2.5. Dynamic stereopsis

**FIGURE 1:**
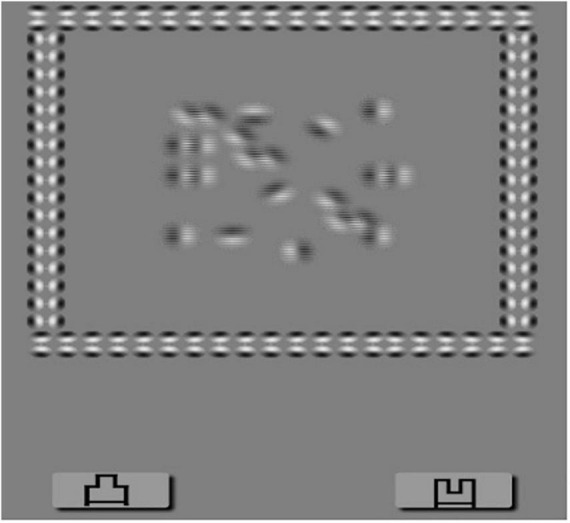
Dynamic stereopsis. Patients wearing 3D polarized glasses are required to recognize that the outlines of letter “N” were elevated from or flat on the screen taking the square as a reference. There are four grades, and the result reach to grade 4 can be recorded as normal.

All subjects wore polarized glasses after refractive correction to observe the stimulus on a screen with a gray background (44 cd/m^2^). Stimulus was a square containing 16 Gobar spots generated by a random-dot kinematogram (RDK) algorithm with a monitor frame rate of 10 Hz. Gobar spots formed two outlines of the letter “N” according to the motion definition structure and were displayed to the two eyes through the polarized glasses. Two letters had binocular parallax and could be fused to form stereoacuity. Subjects were asked to use the square as a reference to recognize whether the outlines of the letter “N” were elevated or flat relative to the screen. There are four grades, and the result reaching grade 4 is normal (Figure 1).

### 2.6. Binocular rivalry signal-noise ratio

**FIGURE 2:**
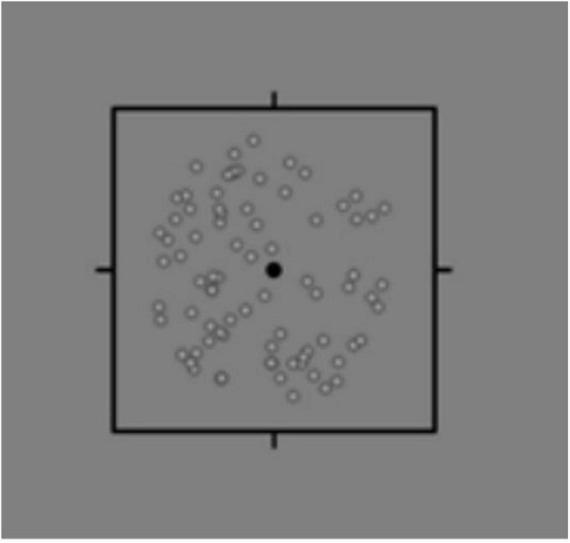
Binocular rivalry signal-noise ratio. Patients wear 3D polarized glasses and watch the moving signal dots and noise dots on the screen. The signal dots moved in all directions uniformly, while the noise points moved erratically. The examinee was required to identify the movement direction of the signal points. After detecting the correct direction each time, the ratio of the signal dots to the noise dots was changed until the patient could not recognize the movement direction of the signal dots; finally, the binocular balance was obtained.

All subjects wore polarized glasses after refractive correction to observe the stimulus on a screen with a gray background (44 cd/m^2^). Stimulus was the moving signal dots and noise dots in the square. Patients were instructed to watch the signal dots with the right eye and the noise dots with the left eye. The signal dots moved in all directions uniformly, while the noise points moved erratically. The examinee was required to identify the movement direction of the signal points. After detecting the correct direction each time, the ratio of the signal dots to the noise dots was changed until the patient could not recognize the movement direction of the signal dots; finally, the binocular balance was obtained. The ratio of signal and noise dots can be divided into eight levels. The ratio of signal dots is 100% at level 1. At level 2, the ratio of signal dots is 85%, and noise dots is 15%. At level 3, the signal and noise dots ratios are 70 and 30%, respectively. Subsequently, the number of signal dots is decreased by 10%, the number of noise dots is increased by 10% at each level, and the ratio of signal dots is 20% at level 8. Each level was tested three times and promoted if it was correct. After examining the right eye, the patient was instructed to follow the signal dots with the left eye, and the right eye should watch the noise dots; we recorded the levels of both eyes, respectively. It is considered normal when the monocular is greater than or equal to grade 6, and the difference between two eyes is less than two grades (Figure 2).

### 2.7. Statistical analysis

Statistical analysis was performed using Statistical Product and Service Solutions (SPSS) (Ver. 23.0.; IBM Corp.; Armonk, NY, USA). The measurement data were expressed as mean ± standard deviation, and the counting data were expressed by rate (%). Comparison of refractive parameters and accommodative functions between emmetropia and myopic in the monocular myopia group were examined by the paired sample *t*-test, and the independent two-sample *t*-test was used to compare the monocular myopia children group and the other two groups, respectively. The comparison between the adolescent monocular myopia group and the other two groups was tested by two independent sample *t*-test. The random dot stereopsis, dynamic stereopsis, and signal-to-noise ratio were analyzed by χ2 test. A *P* value of < 0.05 was considered a statistically significant difference.

## 3. Results

This study comprised 106 patients, 41 (23 men and 18 women) in the monocular myopia children group (Group 1), 26 (9 men and 17 women) in the monocular myopia adult group (Group 2), and 39 (21 men and 18 women) in the binocular low myopia children group (Group 3). The mean age was 10.59 ± 2.24 years (6–15 years), 24.08 ± 1.98 years (19–27 years), and 9.67 ± 2.30 years (6–15 years), respectively. The biometric data of each group are listed in Table 1.

**TABLE 1:** Demographic and biometric measures (mean ± SD) for the subjects’.

	Group 1	Group 2	Group 3
Number	41 (82 eyes)	26 (52 eyes)	39 (78 eyes)
Sex (male/female)	23/18	9/17	21/18
Age (range)	10.59 ± 2.24 (6–15)	24.08 ± 1.98 (19–27)	9.67 ± 2.30 (6–15)
BRD (D)	−1.37 ± 0.93	−1.94 ± 0.91	−0.32 ± 0.27
UCVA (logMAR)	0.25 ± 0.37	0.27 ± 0.42	0.49 ± 0.22
BCVA (logMAR)	−0.11 ± 0.05	−0.12 ± 0.05	−0.11 ± 0.04

Table 2 represents the results of biometric measurements and accommodative functions between myopic and emmetropic eyes in Group 1 and Group 2. There was a significant difference in UCVA (*t* = 14.447, *P* < 0.001), SE (*t* = −9.404, *P* < 0.001), and AA (*t* = 2.108, *P* = 0.041) between myopic and emmetropic eyes in Group 1. There was no significant difference in BCVA (*t* = 0.628, *P* = 0.534) and AF (*t* = 1.372, *P* = 0.178) between myopic and emmetropic eyes in Group 1. In Group 2, there was a significant difference in UCVA (*t* = 12.354, *P* < 0.001), SE (*t* = −10.943, *P* < 0.001), AA (*t* = 2.557, *P* = 0.017), and AF (*t* = 2.131, *P* = 0.043) between myopic and emmetropic eyes but there was no significant difference in BCVA (*t* = −0.717, *P* = 0.480).

**TABLE 2:** Results of biometric measures and accommodative functions between myopic and emmetropic eyes in Group 1 and Group 2.

Group	UCVA (logMAR)	BCVA (logMAR)	SE (D)	AA (D)	AF (cpm)
**Group 1**					
Myopic eyes	0.57 ± 0.26	−0.11 ± 0.05	−1.49 ± 0.78	11.56 ± 2.45	6.16 ± 2.83
Emmetropic eyes	−0.06 ± 0.07	−0.11 ± 0.05	−0.12 ± 0.36	11.04 ± 2.69	5.71 ± 2.87
*t*	14.447	0.628	−9.404	2.108	1.372
*P*	<0.001	0.534	<0.001	0.041	0.178
**Group 2**					
Myopia eyes	0.64 ± 0.28	−0.12 ± 0.05	−1.87 ± 0.79	9.19 ± 1.58	6.69 ± 2.36
Emmetropic eyes	−0.09 ± 0.07	−0.12 ± 0.05	−0.07 ± 0.42	8.58 ± 1.35	5.60 ± 2.45
*t*	12.354	−0.717	−10.943	2.557	2.131
*P*	<0.001	0.480	<0.001	0.017	0.043

**FIGURE 3:**
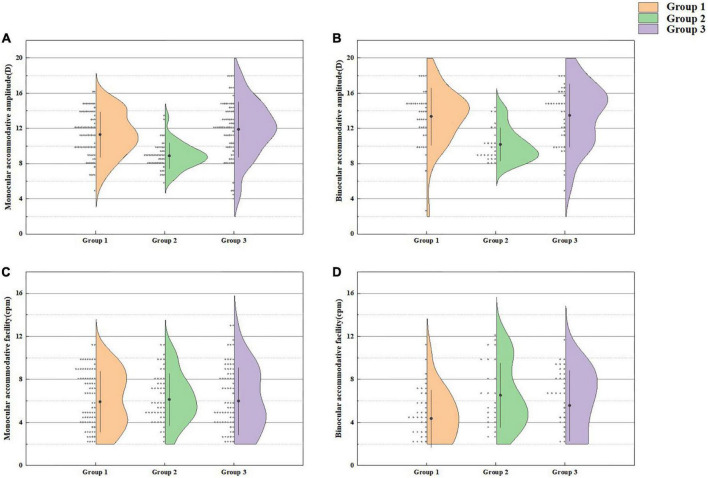
Monocular accommodative amplitude **(A)**, binocular accommodative amplitude **(B)**, monocular accommodative facility **(C)**, and binocular accommodative facility **(D)** in Group 1 and the other two groups.

A comparison of the accommodative functions of Group 1 and the other two groups is presented in Figure 3. There was a significant difference in monocular AA (*t* = 6.879, *P* < 0.001), binocular AA (*t* = 5.043, *P* < 0.001) and binocular AF (*t* = −3.074, *P* = 0.003). Furthermore, there was no significant difference in accommodative functions between Group 1 and Group 3 (all *P* values > 0.05).

**FIGURE 4:**
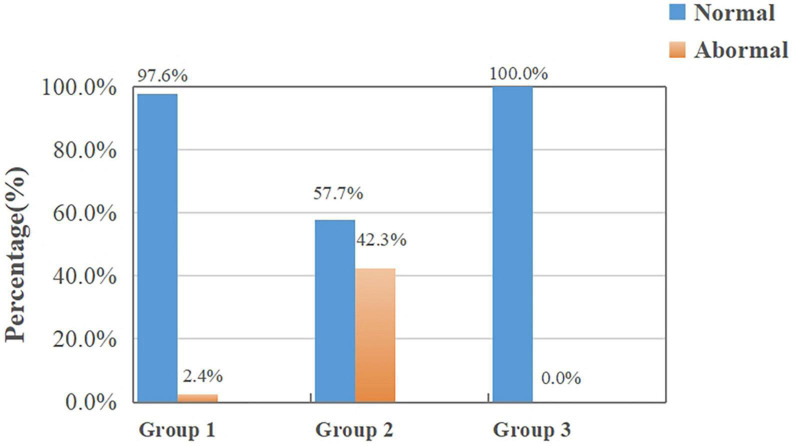
Results of random dots stereopsis in Group 1 and the other two groups.

**FIGURE 5:**
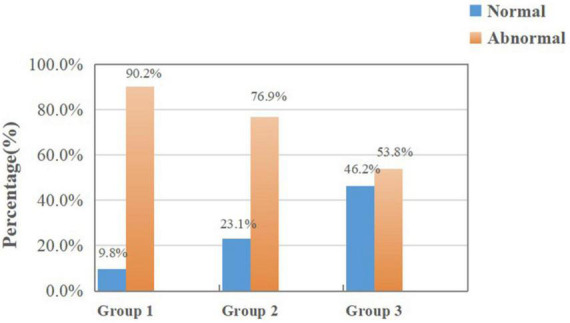
Results of dynamic stereopsis in Group 1 and the other two groups.

**FIGURE 6:**
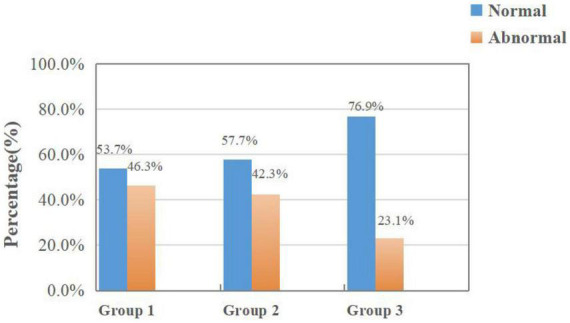
Results of binocular rivalry signal-noise ratio in Group 1 and the other two groups.

Figure 4 shows the results of random dots stereopsis in Group 1, Group 2, and Group 3. The normal rate of random dots stereopsis in Group 1 was higher than in Group 2 (χ2 = 14.596, *P* < 0.001). The results of dynamic stereopsis are given in Figure 5. The normal rate of dynamic stereopsis in Group 1 was lower than in Group 3 (χ2 = 13.281, *P* < 0.001). The results of the binocular rivalry signal-noise ratio are provided in Figure 6. The normal ratio of signal-to-noise ratio in Group 1 was lower than in Group 3 (χ2 = 4.755, *P* = 0.029).

## 5. Discussion

Monocular myopia is very common in clinical practice; such patients, particularly children with monocular myopia, are an indispensable part of the myopic population. Patients with monocular myopia have good vision without glasses and can maintain the needs of daily life (such as writing, reading, and walking). Consequently, many parents and even some adults believe that monocular myopia does not require glasses. They believe using corrective lenses could detract from the beauty, cause inconvenience, and accelerate myopia development. Do patients with monocular myopia need to wear glasses?

However, there is no unified conclusion about the pathogenesis of myopia. Many scholars hypothesized that accommodation is involved in myopia progression (Myrowitz, 2012; Koomson et al., 2016). There is a strong correlation between AA and age (Augusteyn et al., 2011). According to the formula of minimum AA and AF of the corresponding age, the monocular AA and monocular and binocular AF of Group 1 and Group 2 were lower than the normal values. While in Group 3, only the AF was abnormal. These results indicated that the decreased AA and AF might be involved in myopia development. Its mechanism may be that the decreased AA and AF keep the retina in hyperopic defocus for an extended period, thus promoting myopia (Read et al., 2010). Usually, both eyes have symmetrical accommodation. This study analyzed the accommodation function between the two eyes in each group and found no significant difference in AA and accommodative facility between the two eyes in the binocular myopia group. However, in Group 1, the AA of myopic eyes was lower than emmetropic eyes. Furthermore, in Group 2, the AA and AF of the myopic eyes were lower than the emmetropic eyes. These suggested that there was no obvious difference in binocular accommodative function in myopic patients with the same refractive state, but some degree of inequality in the binocular accommodative function of patients with anisometropia was found, consistent with the previous research results (Toor et al., 2019). Hence, this study’s findings proposed that anisometropia could lead to abnormal accommodative function, accelerating the development of anisometropia or binocular myopia.

Previous studies have found a correlation between anisometropia and the progression of binocular myopia (Parssinen, 1990; Tong et al., 2006; Deng and Gwiazda, 2012). Children with monocular myopia may still be monocular myopia in adulthood, which shows that the degree of binocular anisometropia increases, while another part may develop into binocular myopia without obvious pathological anisometropia. Moreover, form deprivation and lens-induced myopia are two classic experimental models of myopia (Bowrey et al., 2015; Fu et al., 2015; Wang et al., 2015). Both models demonstrate that abnormal visual input in one eye could cause the eye axis to lengthen, resulting in a difference in the refractive state of both eyes; thus, an increase in the eye axis is an important reason for myopia progression (Terasaki et al., 2017). First, uncorrected monocular myopia is comparable to form deprivation, causing the eye axis to elongate and accelerate the progression of myopia. Accordingly, some children with monocular myopia may develop greater anisometropia in adulthood. Second, the structural difference influences the refractive state and the efficiency of image processing by the visual pathway, suppressing low-quality images and prioritizing high-quality images (Westendorf et al., 1982; Zheleznyak et al., 1982; Larsson and Holmstrom, 2006; Hashemi et al., 2011). Binocular balance might have a role in this mechanism. Li et al. (2013) and Hess and Thompson (2015) proposed the concept of the binocular balance point and developed a method for detecting the binocular balance point. According to the study’s results, the rate of binocular imbalance in Group 1 was significantly higher than in Group 3. The most important function of vision is to collect external information to guide sports behavior, which necessitates normal visual perception and fine motor control. Binocular visual information depends on the brain’s sensitivity to the spatial and temporal frequencies of binocular retinal images. Therefore, binocular balance can only be achieved when binocular retinal images’ spatial and temporal frequencies are identical (Levi et al., 1979; Vassilev et al., 2002; Vera-Diaz et al., 2018). Binocular vision input by binocular vision imbalance due to strabismus, anisometropia or amblyopia in the early stage (when normal contralateral eyes are used for viewing) could result in eye movement disorders such as unstable gaze and abnormal saccades (Birch et al., 2019). Zhou et al. (2016) also found that the two eyes of patients with anisometropia were significantly imbalanced. The early binocular imbalance may impede the development of the motor area (MT or V5) of the brain, thereby impairing the contralateral eye’s motor processing ability (Zeki, 2015). If the gaze time is prolonged or abnormal saccade may directly affect the visual function, symptoms such as visual dysfunction and fatigue will be produced when performing long-term visual tasks. This influence could be a potential factor for the contralateral eye to become myopic, causing some adolescents with monocular myopia to develop nearsightedness over time. In the progression of myopia, whether it is lens-induced, form deprivation or abnormal accommodation, the clarity of monocular signal input is limited (Kee and Deng, 2008; Siegwart and Norton, 2010). Refractive adaption might be a treatment for binocular imbalance (Zhou et al., 2016), timely correction of monocular myopia can produce consistent images, balance the information processing efficiency in both eyes, and reduce the impact on the normal contralateral eye during visual development.

Stereopsis is an advanced function of binocular vision that refers to the capacity of the visual organs to perceive three-dimensional (3D) space. The index to evaluate stereopsis is the minimum parallax that can be distinguished by both eyes. Stereopsis will be impaired if monocular vision, binocular monocular vision, or binocular fusion are abnormal (Read and Cumming, 2017; Na and Yoo, 2018). Random dots map is a widely used stereopsis detection method in clinical practice. This study showed no significant difference in the random dots’ stereopsis between Group 1 and Group 3, but Group 2 was worse than Group 1. Stereopsis is based on binocular stimulation and fusion and requires the visual perception of the brain’s neural network (Wilson, 2017). With age, more and more neural network connections are formed between the eyes and the brain (Oberer et al., 2018). This structure’s maturity facilitates the maturation of visual functions such as vision and depth perception. During visual development in monocular myopic patients who do not wear corrective lenses, cones and rods of the myopic eye might receive less visual stimulation, and the transmission of nerve impulses from the optic nerve to the visual cortex is also diminished. Under this dual mechanism of binocular competition and inhibition of activation of the cerebral cortex, the fusion function of both eyes deteriorates, thereby diminishing the stereoscopic function in adults (Campos and Enoch, 1980).

Random dots stereopsis is only a sketchy examination in clinic practice. In addition, we used virtual reality platform to detect the dynamic stereopsis of patients more comprehensively. Dynamic stereopsis refers to the difference in the direction, speed and size of binocular retinal images caused by external moving objects, which stimulates the perception of relatively selective neurons in the central direction (Hosokawa et al., 2013; Jain and Zaidi, 2013). The stimulus used in this study was a random and deformable 3D shape composed of binocular parallax. This design allowed us to detect and quantify the parallax in the movement process, equivalent to a simplified version of the real-world object and more consistent with daily life scenarios. Our results showed that all three groups were impaired in dynamic stereopsis, and the normal rate of dynamic stereopsis of Group 1 was lower than Group 3. Thus, it can be suggested that myopic children may result in abnormal visual function in the early stage; however, there are no widely utilized clinical detection tools. More advanced and sensitive methods are required. The dynamic stereopsis of children with monocular myopia is worse than that of binocular low myopia. We think that asymmetric signal input has a greater influence on stereopsis. To some extent, the imaging quality of the myopic retina is blurred in symmetry, and there are still the same amount of visual nerve impulses in the visual cortex in the state of refractive correction, so it has a slight impact on stereopsis. However, the asymmetric signal input in monocular myopic patients has a greater impact on stereopsis. Stereopsis affects our observation of definition, distance, and contrast (Vedamurthy et al., 2016). Once the monocular myopia detection, corrective glasses should be used to correct it in its early stages and maintain the same level of visual signal stimulation.

This study was limited by the fact that in the adult control group, only patients with monocular myopia were chosen. In fact, many children who have recently developed monocular myopia will eventually develop myopia in both eyes. Therefore, future research must include additional samples to demonstrate that uncorrected monocular myopia may contribute to the development of binocular myopia.

In conclusion, monocular myopia could lead to abnormal accommodative function and unbalanced input of binocular visual signals, accelerating myopia. In addition, monocular myopia may cause stereopsis dysfunction, and long-term uncorrected monocular myopia may impair stereopsis acuity in adulthood. Furthermore, patients with monocular myopia may have abnormal stereopsis at an early stage. The commonly used random dots stereopsis test could not reflect the stereopsis in real time. Hence, it is recommended to adopt dynamic stereopsis detection. Generally speaking, children with monocular myopia must wear glasses for timely correction and to rebuild binocular balance and binocular vision function, and delay the development of myopia.

## Data availability statement

The raw data supporting the conclusions of this article will be made available by the authors, without undue reservation.

## Ethics statement

The studies involving human participants were reviewed and approved by the Ethics Committee of the Xiangya Hospital of Central South University. Written informed consent to participate in this study was provided by the participants’ legal guardian/next of kin.

## Author contributions

DW and AX conceived and designed the study. AX, KD, and QF performed the experiments. AX wrote the main manuscript text. YZ and LZ prepared the figures and tables. DW revised the manuscript. LY provided technical support. All authors read and approved the manuscript.
